# Progressive neurologic disorder: Initial manifestation of hemophagocytic lymphohistiocytosis

**DOI:** 10.1212/WNL.0000000000002729

**Published:** 2016-05-31

**Authors:** Claire Murphy, Sira Nanthapisal, Kimberly Gilmour, Sue Laurent, Felice D'Arco, Cheryl Hemingway, Paul Brogan, Despina Eleftheriou

**Affiliations:** From the Infection, Immunity, Inflammation and Physiological Medicine Programme (C.M., S.N., P.B., D.E.), University College London Institute of Child Health, UK; Faculty of Medicine (S.N.), Thammasat University, Thailand; Immunology (K.G.), Neuroradiology Department (F.D.), and Neurology Department (C.H.), Great Ormond Street Hospital NHS Foundation Trust, London; and Paediatric Department (S.L.), Royal Free Hospital NHS Foundation Trust, London, UK.

Hemophagocytic lymphohistiocytosis (HLH) is a syndrome of pathologic immune activation occurring as a primary genetic disorder, or in association with infections, malignancies, or rheumatologic disorders (secondary HLH).^1^ CNS inflammation has been observed in both forms of HLH, but has not been previously reported as the presenting sole manifestation of the disease.^2^

## Case report.

A 14-year-old Caucasian female presented with a 3-month history of headaches, progressive right-sided convergent squint, dysarthria, gait abnormality with progressive difficulty in climbing up the stairs and being able to stand, and hyperesthesia involving her left arm and leg. She had a long-standing history of pervasive refusal disorder for which she was receiving psychiatric support. Ophthalmology examination revealed a VI cranial nerve palsy and papilledema. She had normal tone, with decreased power in upper and lower limbs and absent reflexes, and no ankle clonus; there was no hepatosplenomegaly and no peripheral lymphadenopathy detected. Brain and spine MRI revealed leptomeningeal enhancement and generalized white matter lesions, in addition to mid–cervical cord contrast-enhancing lesions (figure, A–D). EMG demonstrated motor and sensory neuropathy; sural nerve biopsy showed axonal degeneration with mild inflammatory changes but no vasculitis. Hemoglobin was 14.7 g/dL (reference range [RR] 12–16 g/dL), platelet count 261 × 10^9^/L (RR 150–450 × 10^9^/L), white cell count 5.63 × 10^3^/L (RR 4.0–11.0 × 10^3^/L), alanine transaminase 17 U/L (RR 10–45 U/L), and fibrinogen 2.8 g/L (RR 1.7–4.0 g/L); ferritin was not tested. Oligoclonal bands were positive on CSF examination, suggesting a CNS inflammatory process; other extensive investigations excluded infective, rheumatologic, metabolic, and malignant causes. She was considered to have an unclassified neuroinflammatory disease and was treated empirically with IV immunoglobulin (1 g/kg) and corticosteroids (IV methylprednisolone at 30 mg/kg over 3 days and oral prednisolone 2 mg/kg/d tapered over 8 weeks), with resolution of white matter lesions and improvement of the dysarthria, squint, papilledema, headaches, and hyperesthesia, although her mobility remained impaired despite intense physiotherapy. Thirteen months later, she developed a generalized seizure requiring admission to intensive care, with fever and pancytopenia (hemoglobin at 7.6 g/dL, platelet count 55 × 10^9^/L, and white cell count 3.5 × 10^3^/L). Blood PCR for Epstein-Barr virus was positive at 850,000 copies/mL. She was thought to have viral-induced secondary HLH on the background of a neuroinflammatory syndrome. She was treated with IV methylprednisolone at 30 mg/kg/d for 3 days, oral prednisolone 2 mg/kg/d tapered over 4 months, rituximab (375 mg/m^2^ weekly for 4 weeks), and mycophenolate mofetil (1,200 mg/m^2^/d).

**Figure:**
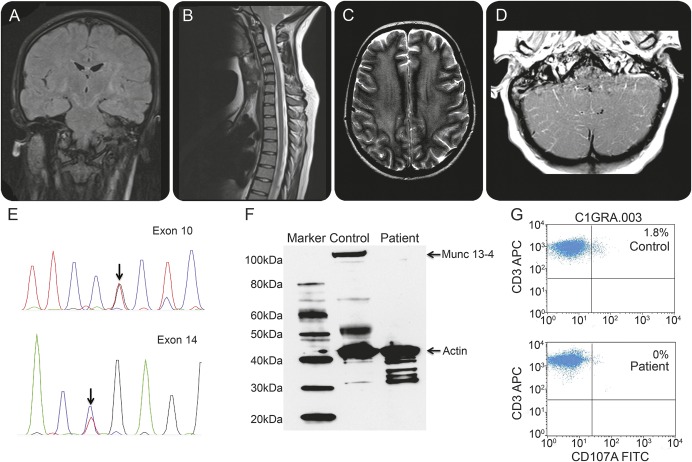
Imaging findings, Sanger sequencing, Munc protein expression, and CD107a degranulation assay in a 14-year-old female with progressive neurologic disorder due to primary hemophagocytic lymphohistiocytosis (A) Coronal fluid-attenuated inversion recovery–weighted MRI of the brain, (B) sagittal T2-weighted imaging of the cervicothoracic spine, and (C) axial T2-weighted imaging of the brain, showing bilateral lesions in the brain (internal capsules, globi pallidi, subthalamic nuclei, deep white matter), as well as mid–cervical spine lesions. (D) Axial postcontrast T1-weighted imaging at the level of the cerebellum revealed bilateral diffuse leptomeningeal enhancement. (E) Sanger sequencing chromatogram of *UNC13D* gene showing compound heterozygous mutations in exon 10 (c.C817T; p.R273X) and exon 14 (c.G1241T; p.R414L) for the same patient. (F) Western blot analysis in patient-derived peripheral blood mononuclear cells shows absence of Munc 13-4 protein relative to a healthy control. (G) Flow cytometric figures show absent percentage increase in CD107a-positive cells in patient-derived CD8 T cells following stimulation with anti-CD3 antibody in relation to a healthy control.

Whole exome sequencing revealed known pathogenic compound heterozygous mutations in *UNC13D*: exon 10 (c.C817T; p.R273X) and exon 14 (c.G1241T; p.R414L),^3,4^ confirmed using Sanger sequencing (figure, E; e-Methods and table e-1 on the *Neurology*® Web site at Neurology.org), Munc 13-4 protein was absent (figure, F), and defective CD8 T cell degranulation was demonstrated (figure, G; e-Methods). Based on these results, the patient was fast-tracked to allogeneic hematopoietic stem cell transplantation. At the time of writing, she remains well with no further hematologic and/or neurologic manifestations of HLH at 4 months after allogeneic hematopoietic stem cell transplantation.

## Discussion.

We report a case of primary HLH that rather atypically presented with an isolated neurologic disorder. We suggest that HLH should be included in the differential diagnosis of unclassified progressive neuroinflammatory disease, even in the absence of pancytopenia. Functional screening assays, such as quantification of T and NK cell degranulation, and intracellular perforin staining, can be used to promptly identify patients with primary HLH and guide management.^5^

Neurologic signs are present in up to 63% of children with primary HLH at disease onset^2^; the majority, however, also have other classic HLH manifestations. Our patient presented with an isolated progressive neurologic disorder, 13 months before more typical HLH features.^2^ CNS involvement in HLH can radiologically mimic neuroinflammatory disorders including multiple sclerosis and acute disseminated encephalomyelitis.^6^ Since diagnostic delay may result in fatalities or irreversible neurologic sequelae,^2^ clinicians should be aware that unclassified neuroinflammatory diseases could evolve into full-blown HLH.

This late development of more typical HLH manifestations in our patient is intriguing. The heterozygous R414L mutation has been described in 2 patients with compound heterozygous mutations in *UNC13D* with late-onset primary HLH associated with encephalopathy in the context of other typical features of HLH.^3^ Patients with at least one allele bearing a nonsynonymous rather than a disruptive mutation may develop symptoms at a significantly older age, suggesting that residual degranulation function allows some defensive capacity.^7^

Our case report also highlights the utility of next-generation sequencing in making early genetic diagnoses and enabling targeted treatments.^7^

## Supplementary Material

Data Supplement
