# Supplementary material for: Progressive neurologic disorder: Initial manifestation of hemophagocytic lymphohistiocytosis
Source: Neurology. 2016 May 31;86(22):2109–11. doi: 10.1212/WNL.0000000000002729 (PMC4891214; doi:10.1212/WNL.0000000000002729)
Supplement: Data Supplement [file supp_86_22_2109__index.html]

Data Supplement 

# Progressive neurologic disorder: Initial manifestation of hemophagocytic lymphohistiocytosis

## Data Supplement

One table and methods; one Microsoft Word file.

**Neurology® data supplements are not copyedited before publication. Published editorials and translations have been copyedited.  
 © 2016 American Academy of Neurology.  
  
 Files in this Data Supplement:**

- Table e-1, e-Methods - Microsoft Word file
