# Supplementary material for: Progressive neurologic disorder: Initial manifestation of hemophagocytic lymphohistiocytosis
Source: Neurology. 2016 May 31;86(22):2109–11. doi: 10.1212/WNL.0000000000002729 (PMC4891214; doi:10.1212/WNL.0000000000002729)
Supplement: Data Supplement [file supp_WNL.0000000000002729_Supplemental_data.docx]

**Supplemental Data**

**Genetic Analysis**

Whole exome sequencing (WES) was completed using 50ng of genomic DNA extracted from peripheral blood of this case. WES library preparation was completed using Nextera rapid capture exome library preparation kit (Illumina), as per manufacturers instructions, and sequencing was completed using HiSeq 1000 platform (Illumina). Sequence data were mapped using Galaxy (1-3) and Human Genome Reference Consortium build 37/hg19 (GRCh37/hg19) as a reference. Over 95% of reads were aligned to the reference genome. SNPs were called using wANNOVAR (4).

Sanger sequencing of *UNC13D* was performed using the primers shown in table e-1. FastStart PCR master mix (Roche) was used for amplification and BigDye Terminator V3.1 cycle sequencing kit (Applied Biosystem) was used for the sequencing reaction. Sanger sequencing was completed using Applied Biosystem 3730 DNA Analyser and calls were made using Applied Biosystem 5.2 software. The sequencing output was analysed using CodonCode Aligned 5.1 to align and visualise the reads.

**Munc13-4 Immunoblot**

Lysates were prepared and immunoblot performed as described in (5). Briefly, peripheral blood mononuclear cells were separated by ficoll paque density gradient separation. Blood was diluted with an equal volume RPMI 1640 (Invitrogen) and layered over lymphoprep (Axis Shield) and centrifuged for 10 minutes at 2500rpm, brake off. Mononuclear cells were collected from interface between lymphoprep and plasma/RPMI layers and washed with RPMI centrifuged at room temperature for 7 minutes at 1500rpm, brake on. Cell pellet was resuspended in NP40 lysis buffer (Ameresco) incubated on ice for 5 minutes and the 2X SDS protein loading buffer (National Diagnostics) was added in equal volume. Lysates were separated on a 4-12% gradient gel (Invitrogen) in MOPs buffer (Invitrogen) and semi-dry transferred to nitrocellulose (Millipore) according to manufacturer’s directions. The nitrocellulose was blocked in 2.5% milk in PBS-Tween (blocking buffer), munc13-4 antibody (AbCam) was added at 1:1000 in the blocking buffer and incubated for 3 hours at room temperature, washed 5 times with PBS-T, fresh blocking buffer was added and anti- goat HRP antibody (Santa Cruz) was added at 1:1000 for 45 minutes, the membrane was then washed 5x and imaged using ECL enhance (GE Healthcare) and imaged on a Genegnome (Syngene Bio Imaging) imager for 1-5 minutes. Post imaging the membrane was washed and incubated with anti-β-actin antibody (Sigma) in blocking buffer and the membrane was processed as above using anti-mouse HRP (Dako) as the secondary antibody.

**CD107a Degranulation Assay**

Peripheral blood mononuclear cells were separated by ficoll paque density gradient separation. Briefly, blood was diluted with an equal volume RPMI 1640 (Invitrogen) and layered over lymphoprep (Axis Shield) and centrifuged for 10 minutes at 2500rpm, brake off. Mononuclear cells were collected from interface between lymphoprep and plasma/RPMI layers and washed with RPMI centrifuged at room temperature for 7 minutes at 1500rpm, brake on. Cell pellet was resuspended in RPMI with 10% FCS to a concentration of 2x10^6^ cells/ml (6).

PBMC were stimulated with interleukin 2 (Chiron) overnight, divided into three separate samples and incubated with fluorescein isothiocyanate-conjugated anti-CD107a antibody (BD Pharmingen) alone (control sample), or stimulated with anti-CD3 antibody (CLB(Mast)) or phytohaemagglutinin (PHA) (BioStat) for 2 hours at 37°C. Samples were then subject to flow cytometric analysis and gated on lymphocytes using forward/side scatter. CD107a expression was analysed on cytotoxic T cells (CD8+, CD3+) and on natural killer (NK) cells (CD56+, CD3) and the percentage increase in CD107a positive cells between unstimulated control sample and samples stimulated with either anti-CD3 antibody or PHA was calculated (6).

**Table e-1** Primers for amplification of UNC13D Exon 10 and Exon 14

| Primer | Forward | Reverse |
| --- | --- | --- |
| Exon 10 | TCTGTGTCCTCACAAAGCTGAG | AATGAGGCCTCTGTGAGCAG |
| Exon 14 | GTCTAGGCCCCTAAAGCATCTC | CTCACTCCTCCAACAATTCTCC |

**References**

1. Goecks J, Nekrutenko A, Taylor J, et al. Galaxy T. Galaxy: a comprehensive approach for supporting accessible, reproducible, and transparent computational research in the life sciences. Genome Biol 2010;11:R86.
2. Blankenberg D, Von Kuster G, Coraor N, Ananda G, Lazarus R, Mangan M, et al. Galaxy: a web-based genome analysis tool for experimentalists. Curr Protoc Mol Biol 2010;Chapter 19:Unit 19 0 1-21.
3. Giardine B, Riemer C, Hardison RC, Burhans R, Elnitski L, Shah P, et al. Galaxy: a platform for interactive large-scale genome analysis. Genome Res 2005;15:1451-5.
4. Wang K, Li M, Hakonarson H. ANNOVAR: functional annotation of genetic variants from high-throughput sequencing data. Nucleic Acids Res 2010;38:e164.
5. Qasim W, Gilmour K, Heath S, et al. Protein assays for diagnosis of Wiskott Aldrich syndrome and X-linked thrombocytopenia. Br J Haematol 2001;113:861-865
6. Wheeler R, Cale C, Cetica V, et al. A novel assay for investigation of suspected familial haemophagocytic lymphohistiocytosis. Br J Haematol 2010;150:727-730
